# Visible and invisible cultural patterns influencing women’s use of maternal health services among Igala women in Nigeria: a focused ethnographic study

**DOI:** 10.1186/s12889-025-21275-9

**Published:** 2025-01-13

**Authors:** Uchechi Clara Opara, Peace Njideka Iheanacho, Pammla Petrucka

**Affiliations:** 1 College of Nursing, University of Saskatchewan, Health Science Building-1A10, 107 Wiggins Road, Box 6, Saskatoon, SK, Saskatchewan S7N 5E5 Canada; 2 Department of Nursing Sciences, University of Nigeria, Enugu Campus, Enugu State Nigeria

**Keywords:** Culture, Beliefs, Practices, Norms, Qualitative research, Maternal health services, PEN 3 cultural model, Nigeria

## Abstract

**Background:**

Explicit and implicit cultural patterns are critical cultural norms, beliefs, and practices that determine women’s health-seeking behaviour. These cultural patterns could limit women’s use of maternal health services, resulting in maternal health complications. The study aims to provide an in-depth understanding of explicit and implicit cultural patterns, their meanings and how they influence women’s use of maternal health services among Igala women in Nigeria.

**Methods:**

Roper and Shapira’s (2000) focused ethnography was employed with 43 women aged 18–43 years recruited using the purposive and snowballing technique. The study was conducted with 21 women for one-on-one interviews and two focus group discussions with six women in the rural area and seven women in the urban area. In addition, participant observation of nine women from the third trimester to birth was conducted, yielding 189 h of observation in two primary healthcare facilities in rural and urban areas. Data analysis was conducted using Roper and Shapira’s (2000) method.

**Result:**

Three themes were generated using the PEN3 cultural model: perceptions, enabler, and nurturers. Subthemes generated under the theme of perception were, Belief in witchcraft, Pregnancy announces itself, I cannot tell people I am in labour, and Unspoken acquiescence to the culture. Under the theme of nurturer, Home birth was found to limit access to maternal health services. The theme of enabler yielded subthemes such as You are not woman enough; I want my placenta and Rising matriarchs.

**Conclusion:**

Implicit and explicit cultural patterns significantly influence women’s use of maternal health services. Given the complexity of culture and its influence on women’s use of maternal health services, multifaceted strategies tailored to the cultural needs of communities are needed to enhance the realization of Sustainable Development Goal #3:1 in Nigeria.

**Supplementary Information:**

The online version contains supplementary material available at 10.1186/s12889-025-21275-9.

## Introduction

Cultural norms, beliefs, and practices are significant determinants of health that influence maternal health decisions and health-seeking behaviours and practices [1, 2]. According to the World Health Organization (WHO) [3], culture is defined as “the set of distinctive spiritual, material, intellectual and emotional features of society or a social group … [which] encompasses, in addition to art and literature, lifestyles, ways of living together, value systems, traditions and beliefs” (p.1). Culture is not only limited to a community’s explicit religious or ethnic beliefs and practices but also encompasses implicit traditional norms that are taken for granted and inform decisions women make in childbirth [3]. These cultural norms, beliefs, and values influence the adoption of practices during pregnancy, birth and postpartum that could predispose women to maternal health complications and death [1, 4, 5].

According to Hall [6], explicit cultural features are values, norms, and practices easily seen or recognized by others outside the group, such as dressing, festivals, and rituals that are amenable to change. However, most implicit cultural values are unconscious behavioural patterns, assumptions, and beliefs rarely shared with other groups and resistant to change [6–8]. Adherence to cultural norms and values is predominant in most sub-Saharan Africa (SSA) [9, 10], where incidentally, 70% of global maternal mortality occurs [2].

While maternal health strategies such as the sustainable development goal (SDG) #3:1 have been developed to reduce global maternal mortality to less than 70 per 100,000 live births, this strategy lacks cultural approaches to address women’s cultural needs and challenges around childbirth [11–13]. Thus, in SSA, maternal health has not significantly improved as the lifetime risk of maternal mortality, which is the likelihood of death from maternal health issues, is 1 in 41 in SSA compared to 1 in 11,000 in most developed countries [2].

As an SSA country, Nigeria is multi-ethnic and multi-linguistic, with over 250 ethnic groups with distinct implicit and explicit cultural norms, values, rituals, and practices significantly influencing women’s use of maternal health services (MHS) [14, 15]. Such influence is reflected in the high maternal mortality index of 1047 per 100,000 live births, placing Nigeria as the third-highest contributor to maternal mortality in 2023 [16]. In Nigeria, while 60% of women attend antenatal care, approximately 50.7% use facility care [17, 18], though this statistic is lower in rural areas with limited access to health facilities. The remaining percentage seek assistance from alternative care systems or unskilled care personnel who lack knowledge and skill on approaches to managing pregnancy and birth, which could result in severe maternal morbidity and mortality [19, 20]. Several maternal health strategies and policies have been developed in Nigeria to address maternal mortality, such as the Abiye program and the Midwives Service Scheme (MSS) [21, 22]. However, these policies have had limited influence in reducing maternal mortality as both are biomedically centred with limited cultural strategies to address several cultural challenges around childbirth.

While childbirth is biological, the sequel of pregnancy and birth are socially constructed and shaped by cultural perceptions and practices [23, 24]. Motherhood is a unique experience shaped by various values and norms, which are beneficial, harmful, and existential, believed to preserve and protect the mother and baby [9, 25, 26]. Several authors have emphasized that the biomedical viewpoint or notion of pregnancy and childbirth is, in most cases, oblivious to the significance of cultural and traditional beliefs and practices surrounding women’s use of MHS [13, 27]. For example, people’s perceptions and views regarding pregnancy and birth are not always shared within Western health facilities. Therefore, maternal health issues may not easily be diagnosed, given conflicting viewpoints which delay women’s access to facility care while favouring traditional and alternative healers [28]. Thus, disregarding culture in care provision not only leads to an emphasis on biological well-being as a single determinant of health [13] but also fails to promote an understanding and strategies of how culture could be integrated as a significant factor in health promotion, especially among populations where there are limited or no formal health facilities [8].

While many studies have identified culture as a contributor to maternal mortality in Nigeria, limited studies have aimed to provide a deep understanding of diverse cultural factors, their meaning and how such cultural norms influence women’s use of MHS [14, 20, 29]. Moreover, no such studies have been conducted among the Igala ethnicity, which has a rich cultural heritage that influences most decisions around childbirth. Consequently, understanding these implicit and explicit cultural patterns among women who have experienced pregnancy and birth could foster appropriate cultural maternal health interventions to enhance maternal health outcomes. To promote an in-depth understanding of these norms, the PEN 3 cultural model was employed as a theoretical framework for the findings in this study. The PEN-3 cultural model is a framework that contextualizes the role of culture in shaping understanding of health beliefs and practices, which has been used in several countries, including Nigeria, where the study was conducted [30–32].

## The PEN-3 cultural model

The PEN-3 cultural model is a framework that allows for exploring and understanding contextual and cultural issues that influence health behaviour. The PEN-3 consists of three interrelated domains, each with three PEN acronyms, namely Cultural Identity (person, extended family, neighbourhood), (2) Relationships and Expectations (perceptions, enablers, and nurturers), and (3) Cultural Empowerment (positive, existential, and negative) [33]. The cultural identity domain features the entry level of interventions that could occur through persons (mothers or health care workers), extended family (grandmothers), or neighbourhoods (communities or villages) [34]. The relationships and expectations domain also forms a part of the assessment phase, where people’s perceptions, attitudes and behaviours concerning a health problem are explored [35]. The structural resources, health systems, and family influence are also explored to determine how these factors influence effective health-seeking practices [33]. The second assessment phase, the cultural empowerment domain, explores health issues to understand those that are positive, existential, or harmful to health [34]. This study used the relationships and expectations domain to explore explicit and implicit cultural factors that influence women’s use of MHS.

### Aim

To explore the meaning of explicit and implicit cultural factors and how they influence women’s use of MHS among the Igalas in Nigeria.

## Design

This study was conducted using the focused ethnographic (FE) methodology described by Roper and Shapira [36]. Roper and Shapira’s [36] FE focuses on exploring a subculture of a population using a predetermined research question within a limited period. FE aims to understand cultural phenomena and how cultural constructs influence health behaviour. Findings from the research could promote the provision of culturally appropriate health services to enhance maternal health outcomes. The FE described by Roper and Shapira [36] was deemed appropriate for this study as it allowed us to explore and understand implicit and explicit cultural beliefs, values, practices, and experiences using diverse methods such as participant observations, interviews, focus group discussions, reflective and field notes within a naturalistic environment. Using participant observation over time enhanced rapport and trust between participants and the researcher, allowing participants to freely share their experiences with the researcher. In addition, participant observation provided a deep insight into participants’ actions and behaviours and the cultural dynamics within the social context. Using the FE by Roper and Shapira also allowed the researcher to conduct semi-structured interviews and focus group discussions, which promoted a rich exploration of the cultural beliefs and practices influencing the use of MHS among Igala women. These diverse methods allowed for the integration of emic (participants’ experiences) and etic (researchers’ knowledge and experience), which promoted a thick description and interpretation of collective cultural norms and practices influencing the use of MHS among the Igalas in Kogi East Nigeria.

Considering that Roper and Shapira’s [36] FE emphasizes the need for researchers to have a theoretical knowledge of the phenomenon understudy and a knowledge of the population of interest, all the authors are nurse research scholars with in-depth knowledge of the phenomenon of interest and the research methodology. Though the third author is unfamiliar with the research context, the first and second researchers have lived and worked among the Igalas for over 20 years. This study forms part of a larger doctoral research project to explore the cultural beliefs and practices influencing women’s use of MHS in Nigeria. The Standard for Reporting Qualitative Research (SRQR) guided the reporting of this study [37].

### Positionality and reflexivity

All the authors’ beliefs, values and theoretical orientation are rooted in the interpretive paradigm, which is the lens through which we see the world, reflected in this study’s ontological, epistemological, axiological and methodological underpinning. Our ontological belief in multiple realities enhanced our understanding of the research question, the phenomenon of interest and the approaches we adopted to explore the research problem [38]. Our epistemological lens of social constructivism also influences the choice of FE as a methodology, the diverse methods we employed, and our approach to data analysis to enhance a thick description of the phenomenon under review [36, 38]. We believe the FE methodology is value-ridden based on axiological underpinning, considering that the researchers’ preconceptions, beliefs and knowledge could influence the data collection and analysis process [36]. Consequently, reflexivity was a vital aspect of the research that allowed each researcher to examine and continuously question our taken-for-granted beliefs, judgements and assumptions throughout the research period [39]. The research paradigm was also instrumental in choosing an appropriate research methodology that allowed for a rich description and interpretation of women’s experiences.

The first researcher collected the data, and so due to her exposure to the research population, reflexivity was an important criterion that challenged her understanding of several cultural beliefs and practices around childbirth to reduce bias [36, 40]. Though the first author’s background as a nurse enhanced access to the participants, the researcher was unfamiliar with the study context, health personnel and routine of work, which promoted the desire to learn from the population and enhanced reflexivity in participant observations [36]. A continuous debriefing with members of the research team also assisted in challenging the first author’s assumptions and preconceived notions throughout the research process to reduce bias.

### Research setting/ sample

The study was conducted in two primary health care facilities in the rural and urban areas of Olamaboro and Dekina, local government areas (LGAs), in Kogi State, Nigeria. The rural facility used for this study was in Olamaboro LGA, with a population of approximately 213,900 and an estimated population of 352,300 in Dekina LGA [41]. Each facility is managed by community healthcare workers and other ancillary staff such as laboratory assistants, storekeepers, pharmacy attendants and cleaners. These facilities were chosen due to their comprehensive health services, encompassing antenatal care, birth services, treatment of uncomplicated tropical health issues, immunization services and antiretroviral treatment.

The participants were Igala women of reproductive age of 18–43 years who were pregnant and attended antenatal care or have given birth in Kogi State primary health care facility within the last 12 months. Igala women who delivered at home in the previous 12 months were also included in the study. We excluded women of other ethnicities in Nigeria to ensure we meet the criteria of focused ethnography that focuses on a subculture of a population. We employed the purposive sampling technique to ensure participants were women who had experience with the desired phenomenon of interest. The snowballing technique was also employed when participants referred other women who met the inclusion criteria. See Table 1 for the characteristics of participants in the study.

**Table 1:** Participants demographic characteristics

	Number	Age	Marital status	Parity	Educational qualifications	Religion	Occupation
Rural Area Participant Observation (RA/PO),	*n* = 5	20–33	Married	1–4	High school diploma (5)	Christians (2) Muslims (3)	Housewives (5)
Urban Area Participant Observation (UA/PO),	*n* = 4	24–27	Married	1–4	Nil education (2) High school diploma (2)	Muslim (3) Christians (1)	Housewives (4)
Rural Area Interviews (RA/IDI),	*n* = 11	20–43	Married	2–6	Nil education (1) High school diploma (5) Associate degree (4) Bachelor’s degree (1)	Muslim (6) Christians (5)	Housewives (8) Business (1) Teaching (1) Nursing (1)
Urban Area Interviews (UA/IDI),	*n* = 10	19–40	Married	1–5	High school diploma (5) Associate degree (4) Bachelor’s degree (1)	Muslim (6) Christians (4)	Housewives (8) Public servant (1) Teaching (1)
Rural Area Focus Group Discussions (RA/FGD),	*n* = 6	23–42	Married	1–4	High school diploma (2) Associate degree (3) Bachelor’s degree (1)	Muslim (3) Christians (3)	Business (3) Teaching (3)
Urban Area Focus Group Discussions (UA/FGD)	*n* = 7	18–43	Married	1–5	High school diploma (4) Associate degree (2) Bachelor’s degree (1)	Muslim (6) Christians (1)	Business (3) Teaching (1) Public servant (2) Farmer (1)

**Definitions** Rural Area Participant Observation -(RA/PO), Urban Area Participant Observation- (UA/PO), Rural Area Interviews –(RA/IDI), Urban Area

Interviews -(UA/IDI), Rural Area Focus Group Discussions -(RA/FGD), Urban Area Focus Group Discussions –(UA/FGD).

### Data collection

We employed several data collection methods, such as participant observations, interviews, field note, reflective or memo note, and focus group discussions [36] between August and November 2023. The recruitment for the selective observations ran concurrently with the one-on-one interviews and focus group discussions. The focus group discussions were employed as a study triangulation conducted in each facility towards the end of the study. These methods enhanced the depth, thickness and interpretation of our findings.

### Participant observations

 Two types of participant observations were employed in the study. The researcher began with passive observation, which allowed for understanding processes and relationships in the research context. Later, selective participant observation was employed, facilitating the observation of nine women in the third trimester of pregnancy and four of these women in labour and birth. Observation flyers were posted at strategic areas in both health facilities to ensure individuals were aware of such observations. Participant observation was accompanied by an observation guide that aimed at understanding women’s cultural needs and challenges around pregnancy and birth, address of these cultural needs by care providers during antenatal health talk, birth and postnatal health talks, women’s cultural preferences and how such were respected around pregnancy and childbirth, quality of culturally appropriate care provided, approach to communication, verbal and non-verbal cues. Each observation lasted 4–5 h but lasted longer on days the researcher was observing women in labour. The total hours of observation were 189 h. All observations were recorded in a field note daily and converted as a Microsoft Word™ document within 12 h of observation to enhance accuracy. A reflective note was also used to document the researcher’s feelings and assumptions related to the explored and observed phenomenon.

### Interviews and focus group discussions

The first author gained access to participants during antenatal health talks and child immunization schedules through the assistance of a gatekeeper in each facility, who invited the researcher to share talks about her research and invite women to participate in the study. Women interested in participating in the research met with the researcher privately after the health talk and immunization schedule, and the researcher explained the aim and purpose of the study. Women who showed interest were told to obtain oral consent from their husbands before signing the consent form to participate in the study. The first author conducted all interviews and focus group discussions between August and November 2023. The interview guides for the study were self-developed by the authors based on the study objectives and validated by two authors to ensure the questions were congruent with the research aim and purpose. See the supplementary file for interview guides used for the study. All interviews were conducted in safe places for the participants and the researcher and lasted 30–50 min.

A total of eleven women from rural areas and ten women from urban areas participated in the study. Semi-structured questions guided the interview. The main questions included: What MHS are provided in this facility? Can you tell me about cultural beliefs and practices influencing your use of MHS? How do these beliefs, values, and practices influence when and how you use MHS? What do these cultural beliefs and practices mean to you as an Igala woman? What is your view on traditions and norms influencing the use of MHS? When do you register for antenatal care in this facility when you notice you are pregnant? What advice do you have for women regarding cultural norms and practices that influence women’s use of facility care?

To enhance the triangulation of our findings, we conducted two focus group discussions in the two health facilities with six women in rural areas and seven women in urban areas who were either pregnant or had recently given birth in each research context. The first author conducted the focus group discussions in a safe environment with the participants alone, which lasted an average of one hour and 15 min and was guided by the same guide used for interviews.

Oral consent to participate in the study and audio record of the narratives were obtained from participants in the interviews and focus group discussions. Interviews and focus group discussions were primarily conducted in English and a few with a mix of English and Pidgin English (an Indigenous language of communication derived from the English language), spoken generally in Nigeria and by the first and second authors. A back translation of all interview guides, consent forms, and recruitment documents was performed by the first author and validated by the second author, who also speaks pidgin English. Audio files of the interviews and focus group discussions conducted in English were transcribed verbatim. In contrast, files conducted in pidgin English were transcribed verbatim in pidgin English by the first author and, transcribed into English by the first author and validated by the second author. The research team approved the final version at regular debriefing meetings.

In this study, insight was gained from the early observations and interviews, which then informed later observations and interviews. The researchers kept moving back and forth in observations and interviews, generating additional insights and ideas to create a detailed description and interpretation of themes and subthemes generated in the study. Thus, we reached data saturation in this study when generated themes were supported with significant data with no new or contradictory information.

### Data analysis

Data analysis was iterative and started with data collection following a back-and-forth movement until data saturation was achieved. Data analysis was conducted manually, which enhanced researchers’ immersion in the generated data throughout the research process. The five steps of data analysis by Roper and Shapira [36], namely coding for descriptive labels, sorting for patterns, identification of outliers or negative cases, generalizing with constructs and theories, and memoing and reflective remarks, were employed in this study. However, these steps were not followed serially, as the researchers kept moving back and forth through the epistemic pole of knowledge creation until a thick description and interpretation were achieved [42, 43].

In the first step of coding for descriptive labels, the researchers read the transcripts line by line repeatedly and met regularly for debriefing to get an overview of the broad themes and patterns running through the data. The authors met regularly to discuss data coding and understand the implicit and explicit cultural patterns and how they influence women’s use of MHS. The second step of sorting for patterns allowed the researchers to immerse themselves in the data through repeated reading of the transcripts to understand the subthemes generated as well as the categories and patterns of such subthemes. These generated themes were then compared with interviews and observations as the research team continued to meet with a continued generation of new themes and subthemes, requiring further exploration of evolving themes. In the third step of identification of an outlier or negative cases, the researchers sought to understand generated themes unrelated to the research question, which are noted and observed to see how such themes influence cultural patterns influencing women’s use of MHS. In the fourth step of generalizing of constructs and theories, the researchers sought to understand how generated themes relate to existing literature. The last step of memoing and remarks ensured researchers’ continued reflection and transparency using a reflective note documented throughout the research process [40]. See Table 2 for themes, codes, and excerpts of the study findings.

**Table 2:** Themes subthemes and excerpts

Themes	Subthemes	Excerpts
**Perceptions**	**Belief in Witchcraft**	I believe the witch…and wizards they are at work every now and then. Sometimes, they will hold some women at home and say don’t go to hospital. They will be talking inside of them, sometimes they will even come in person advising them, “do you want to go to hospital, don’t go”! “If you go, they will give you some drug that will miscarry your baby, that it will lead to abortion (RA/IDI/04).
	**Pregnancy Announces Itself**	…during the first pregnancy, in fact during the first months, you ought not to disclose the fact that you are pregnant until the pregnancy is visible to people. There is that belief that it kind of guides you from evil eyes; people will not harm you. By the time your pregnancy is visible to everybody, the stage of harming you is already over (RA/IDI/01). Why they don’t register immediately they get pregnant is because…maybe you came to the hospital with pregnancy of two, three or four months …and on reaching there you will see that your family is there, your relations, your friends …they will know earlier, and you will have problems (RA/IDI/08).
	**I Cannot Tell People I am in Labour**	Some people might hate you, so if you are delivering, they will go spiritual and do something bad over your head for you not to deliver, so that will make you not to tell your relations(UA/IDI/03).
	**Unspoken Acquiescence to the Culture**	Our elders (abo Ogujuo) are respected, and what they say is important we listen to them (RA/Observation/ 01).
**Enabler**	**Home Births**	Truly, when I deliver at home, I am greatly supported by my family and friends… give me my medicine, give me my food, and cook for me for seven days…and serve all my children (UA/IDI/08). People say they do home delivery because they don’t have money. That if you go to the hospital they charge (UA/IDI/03). I deliver at home because, if my labour comes, it will come very easily to me; it does even take 20 to 30 min before I deliver (UA/IDI/08). many labour come out in the night and you came to the hospital, you cannot meet any nurse (RA/FGD/04).
**Nurturers**	**You are not Woman Enough!**	I felt bad… when I am crying some of my doctors came to advise me that I should not cry, that it is the normal way of delivering, but then my mind was not at rest. I was crying because my others are not giving birth in that way (RA/IDI/03). When I heard what they said that you don’t have waist, she went into labor for four days, I said the major thing is that my baby is here and I am alive (RA/IDI/06).
	**I Want My Placenta!**	That is how I disposed of my own when I give birth in the hospital, my husband brings it back to where we live and then buries it there (UA/FGD/01).
	**Rising Matriarchs**	Like me, if I see any of my friends, I will ask them I hope you are attending a good clinic. We don’t want anything to happen to you or your baby. They always laugh and say you are always concerned. Yes, I am concerned; I don’t want anything to happen to my friends (RA/IDI/O1).

### Trustworthiness

The criteria of credibility, dependability, confirmability, and transferability described by Lincoln and Guba [44] were used to enhance rigour and trustworthiness in this study. To enhance credibility, the instrument was piloted on three women with similar attributes to those of the participants to ensure the appropriateness of the instruments to the research aim and purpose. We also employed triangulation, deep immersion in the data, member checking of transcripts with participants and prolonged engagement in the context. We continued to meet as a research team to deliberate on the generated themes to enhance clarity. To enhance dependability, we ensured a detailed description of the research process. Data coding was done by members of the research team to reduce bias. To enhance confirmability, all observations were documented in field notes and entered in a Word ^TM^ document with the time and date of such observation. A detailed reflective note was used throughout the research period. We enhanced transferability by recruiting women with varied demographic attributes to enhance heterogeneous and rich data.

## Results

**Fig. 1:**
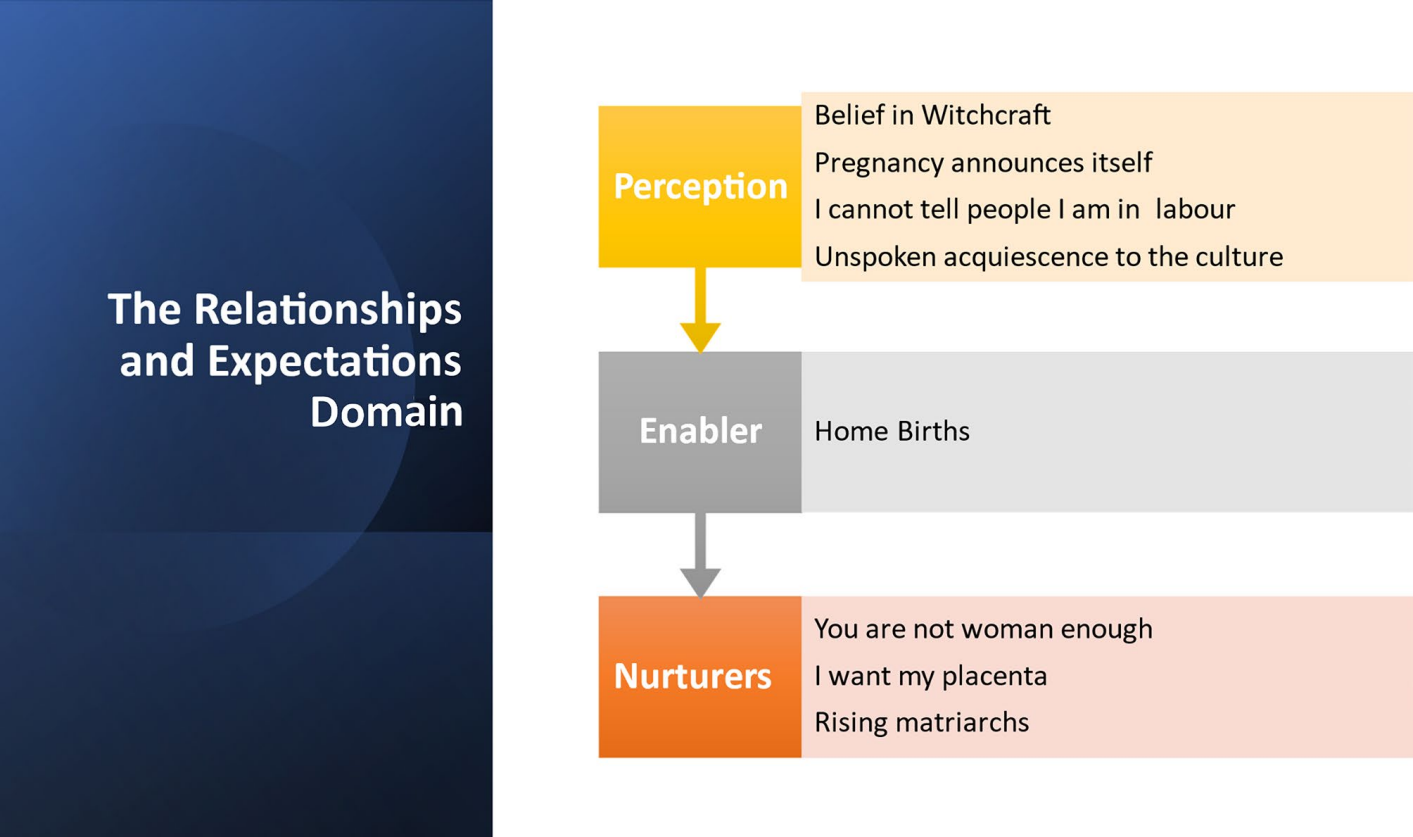
Themes and subthemes using the PEN 3 cultural model

A total of 43 Igala women aged between 18 and 43 years of diverse characteristics in age, parity, education, religion, occupation, and location participated in this study. Using the relationships and expectations domain of the PEN 3 cultural model, three themes were generated from the findings: Perceptions, Enabler and Nurturers. Under the theme of perceptions, we found that Belief in witchcraft was a significant perception that influenced women’s decision-making and limited access to MHS. Additionally, Pregnancy announces itself; I cannot tell people I am in labour and Unspoken acquiescence to the culture also negatively influence women’s use of MHS. Under the theme of enabler, we found that Home birth was a significant factor that limited access to facility care. We also found that themes under nurturers, such as You are not woman enough, I want my placenta, and Rising matriarchs were nurturers which influenced the use of MHS. These themes and subthemes are discussed in this section. See Fig. 1 for Themes and Subthemes generated using the PEN 3 cultural model.

### Perception

The authors explored women’s beliefs, perceptions, assumptions, and values that either enhance or limit the use of MHS. The main themes generated were Belief in witchcraft, Pregnancy announces itself, I cannot tell people I am in labour and Unspoken acquiescence to the culture. These were significant perceptions and values that influenced women’s use of MHS.

#### Belief in witchcraft

A recurring theme in both contexts where data was collected was the deep belief in witchcraft. Women narrated that pregnancy was a time when women were susceptible to attacks from witches and witchcraft from people who they believe possess diabolical powers to harm a pregnant woman or the unborn child. According to the participants, attacks associated with witchcraft always occur during pregnancy, with the early periods of pregnancy being the period where most women can easily become the targets of such attacks. Women recount that such attacks could also come from neighbours, in-laws, relations who appear friendly and even from known enemies. Witchcraft attacks could come while the woman is asleep in the form of masquerades attacking or flogging the pregnant woman, eating in a dream or terrifying nightmares that continuously reoccur. However, some women also narrated that witches can also come in human form to advise women against seeking facility care, though some women may not understand the negative implications of such counsel that could result in maternal and fetal mortality.

It has a lot of influence…I believe the witch…and wizards they are at work every now and then. Sometimes, they will hold some women at home and say don’t go to hospital. They will be talking inside of them, sometimes they will even come in person advising them, “do you want to go to hospital, don’t go”! “If you go, they will give you some drug that cause you to miscarry your baby, that it will lead to abortion.” It is a lie; they are confusing you so that you don’t go…So you might agree with them and stay at home, so that they could have effect in you so that is what I believe. Sometimes they do, and sometimes they might come in your dream; they turn to someone you love. Maybe you might be thinking that maybe it is my mother, it is my mother in-law, it is a lie, maybe someone else, but they are using their face; like me, my fourth pregnancy that I miscarried because I slept in the dream, someone will be coming to take my pant, once they took my pant, I will miscarry the baby, once they gave me red oil in the dream, or pepper or tomato, fresh tomato, I will miscarry it. Sometimes they will come in the form of my own blood sister… it is only God that would help… sometimes you will be dreaming that someone that you love, maybe your mother, your sister, will come to you in your dream, don’t go to hospital, once you go to hospital, the baby will die. It might not be them it might be the witches confusing you, or they might come in human in a daytime like this, don’t go to hospital, this your pregnancy, the way I am seeing it, don’t go to hospital. If you go to hospital, you will die…that is how they confuse women not to go to facility (RA/IDI/04).

Many women believe that attacks from witchcraft could lead to abortions or death of the mother or the baby in the womb, which imposes overwhelming fear in the hearts of many women. Such fear results in women seeking protection from alternative care providers or religious groups and prayer houses, who are believed to provide solution and divine resolution of health challenges, which ordinarily cannot be achieved using Western medicine. According to participants, prayer houses provide care in the form of hibernations for weeks and months during pregnancy, especially if such pregnancies are associated with complications. Women also narrated that prayers, fasting, and the use of holy water and olive oil for prayers also recommended by prayer houses are used to bring lasting solutions to witchcraft attacks around pregnancy and childbirth. For some who seek an alternative care approach, herbal medicines, waist beads or ropes and rituals are recommended for women to ensure protection from witchcraft attacks. However, many women acknowledged that though such witchcraft activities exist, they do not believe in such, as they believe that God, who allowed the pregnancy to happen, can shield them from all negative and evil forces.

#### Pregnancy announces itself

Traditionally, pregnancy in Igala land is usually not announced as people believe that pregnancy should announce itself as the pregnancy advances. Many women believe that such announcements could place the pregnant woman in danger and attract “evil eyes,” evil people who may harm the woman. Women narrate that such attacks occur especially in early pregnancy when it is believed pregnancies are susceptible to attacks from evil forces or witchcraft. Women believe that babies in the early period of pregnancy could easily be transformed into abnormal babies by evil people. Thus, women usually keep a sealed mouth on the status of their pregnancy until such pregnancies reach advanced stage and everyone is aware that they are pregnant. At an advanced stage, women narrate that pregnancies are safe and may not easily be under attack by evil people.

…during the first pregnancy, in fact, during the first months, you ought not to disclose the fact that you are pregnant until the pregnancy is visible to people. There is that belief that it kind of guides you from evil eyes; people will not harm you. By the time your pregnancy is visible to everybody, the stage of harming you is already over (RA/IDI/01).

During the early period of pregnancy, secrecy in disclosing their pregnancy status also extends to limited access to facility care. Women believe that such access could result in people seeing them and spreading the news of their pregnancy throughout the community, which could lead to attacks from evil people and enemies that could affect the mother and baby. Many women also recounted that such early disclosure of pregnancy could result in evil people monitoring women’s pregnancy progress to cause harm during birth. So many women during this period seek advise and assistance from chemist shops or traditional birth attendants who are better trusted on such issues. Moreover, registration in a health facility could be delayed till when a woman is five months or more and the pregnancy is visible to everyone.

Why they don’t register immediately they got pregnant is because…maybe you came to the hospital with pregnancy of two, three or four months …and on reaching there you will see that your family is there, your relations, your friends and you came there, they will know earlier and you will have problems. They will be saying this lady is pregnant to everyone. That we came and met her at the ante-natal. They will continue to say that. That will be a problem because you don’t know who is a bad person… and you will see blood from nowhere, and you don’t know how the blood came about (RA/IDI/08).

Many women provided other reasons for not registering early in the facility when they are pregnant, such as not feeling sick in early pregnancy and the feeling that the baby has not yet formed into a human, so antenatal care may not be needful. However, there remain some women who narrated that they register early for antenatal care during pregnancy because they believe early registration could enhance early detection of abnormalities and complications that are preventable, which, if left unattended to, could result in maternal or fetal mortality.

#### I cannot tell people I am in labour

Women also believe that the period of labour should be kept secret as women are believed to be in a situation where they could easily be susceptible to attacks from evil people. Additionally, women believe that opening up that one is in labour is a foolish act that could lead to severe consequences such as prolonged labour and fetal or maternal mortality due to attacks from evil people. Apart from that, women prefer to only confide in their husbands or mothers when they are in labour as they believe that if friends or extended relations accompany them to a health facility, some of their utterances during labour, which may not be pleasant, could be shared with other people and they will end up being the joke of the community. Consequently, many women narrate that they do not access facility care during labour too early until they are sure they are in established labour and only in most cases with their husbands, mothers or sisters, whom they trust. The news about childbirth is only announced when a woman has safely delivered without associated birth complications.

I will not tell them. Only my husband and I will be in the hospital. I will tell them when I deliver, and they will come to the hospital…Some people might hate you, so if you are delivering, they will go spiritual and do something bad over your head to make you not deliver, so that will make you not tell your relatives (UA/IDI/03).

#### Unspoken acquiescence to the culture

Women narrate that In Igala culture, respect is paramount; therefore, nobody wants to compromise on respect, both the giver and receiver of the respect. This is especially applicable in the matter of respect for the elderly or anyone considered to be in any position of authority over one. Even distant relatives and others who may not be related reasonably can have a say in a person’s matter. Women recount that they are particularly placed at a disadvantage given their position in the culture where they are expected to serve their husbands. Therefore, when a woman’s maternal health-seeking needs happen to be at odds with the elders’ opinions or viewpoints, such needs may have to give way, especially in the form of choice of place to deliver. Most women recounted instances where they or their friends remained quiet in situations where decisions were made about them, even though they were aware that such decisions would not benefit their health to avoid being seen as disrespectful.

In this our culture, we as women cannot take decision alone in Igala land, our husband, our in-laws, our parents too. When they are not around any elder in the family can take that decision. Our elders (abo Ogujuo) are respected and what they say, is important we listen to them. When I was pregnant for my first baby, I started seeing blood, and I was afraid… my husband was not at home, so my father in-law said they should take me to (calls the name of the hospital), but I was using a private hospital where the midwife was taking good care of me…I could not say no to Baba (father-in-law) or tell him I wanted to go to another place… if I do that and they tell my husband, he will not be happy with me…but my baby died, my first son, I was very unhappy, I cried and cried and wanted to also die…You don’t say I don’t want that or want this (RA/Observation/01).

### Enabler

In this construct, we explored structures and systems in the communities and found home birth to be a negative enabling factor within the alternative health system, which limited women’s use of MHS.

#### Home births

Home births were a significant negative enabler generated during the study. Home births happen either in the woman’s home or in the homes of community health attendants and unskilled care attendants working in community health facilities. The most recurring reason for home birth narrated by women was the cost of MHS. Women narrated that births are paid out of pocket by women, some of whom may not afford such costs due to limited socioeconomic status and poverty. Consequently, many women who cannot afford the cost of facility birth usually opt for alternative cheaper options and deliver with traditional birth attendants or unskilled birth attendants in the communities.

People say they do home delivery because they don’t have money. That if you go to the hospital they charge. And the charge for a male child is different from the charge of a female child. That is the main reason why people are drawing back. Another reason is that when you give birth at home, you will just give her a little thing. The money is not up to what they are paying in the hospital. So, they feel that they should give birth to their child at home or maybe a friend of theirs is a midwife or just a woman that has the experience (UA/IDI/03).

Women also narrated that precipitated labour, where most women have rapid labour and deliver within three hours of falling into labour, was a reason why most women deliver at home. Women see such precipitate labour as an act of God. However, for other women, the reason for home birth was as a result of the significant care and support they received at home, which was better than the support provided in most facilities.

Truly, when I deliver at home, I am greatly supported by my family and friends…The following day, they will come back to give my baby a bath and also give me a hot, soothing bath, even when I tell them I can bathe myself. They will say no, we want your body to be very strong and go ahead to prepare the bath, give me my medicine, give me my food, and cook for me for seven days…and serve all my children (UA/IDI/08).

Many participants reported that most home deliveries occur due to lack of access to facilities and health workers when labour starts at night. Most primary health facilities do not provide 24-hour services. Thus, when women go into labour at night, they end up with the option of delivering either in their home or with an unskilled birth attendant. Though home deliveries were common in the communities where data was collected, many women reported that they do not engage in home birth due to the risk of bleeding and other complications after birth, which many unskilled attendants lack the knowledge and capacity to manage.

### Nurturers

We also explored nurturers within communities, such as families and kin, who support women’s use of MHS. Themes such as you are not woman enough; I want my placenta and rising matriarchs were generated under this construct and will be discussed in this section.

#### You are not woman enough

Every woman in the community desires a vaginal birth, which is accepted as a natural birth process, a sign of bravery and a proof that you are a woman. Based on this, many women who end up having a cesarian section are looked down on and are seen as weak women who do not possess the strength to push out a baby, or a “half-human” or women who do not have “a waist” (adequate pelvis) or one under a curse or attack of witchcraft. Due to cesarian section, many women narrate that they have lost the respect of their husbands and in-laws, and their husbands are continually advised to marry women who will have normal deliveries to reduce the money spent on cesarian sections. Due to these interpretations, many women who had cesarian sections narrated that they were depressed and cried for weeks after birth and wondered why they could not achieve a natural birth. Some also sought alternative care options from prayer houses and alternative medicine to achieve a normal vaginal birth with no positive result.

I felt bad… when I am crying some of my doctors came to advise me that I should not cry, that it is the normal way of delivering, but then my mind was not at rest. I was crying because my others are not giving birth in that way. Why is my own case now different? …People were looking at me as if I am not a qualified human being. In this our community, especially if you are passing, some are looking at you as if you are not a complete human being again (RA/IDI/03).

Apart from the dominant cultural interpretations of cesarian section, many women narrated that the fear of death, fear of not achieving a subsequent normal birth, fear of pain, fear of inability to conceive, and fear of the doctor leaving surgical items in the womb, compels them to reject cesarian section and to seek alternate care system during pregnancy. However, cesarian section is accepted by many women who believe that their life and that of the baby are more important than all the name-calling in the communities, as a participant recounted.

When I heard what they said that you don’t have ‘waist’, she went into labor for four days, I said the major thing is that my baby is here and I am alive. I can go anywhere with my baby, and I will see my baby coming for me. That is the major thing. So it is them that have that problem; as for me, I don’t have an issue with them (RA/IDI/06).

#### I want my placenta!

The cultural interpretations and value of the placenta was also a significant subtheme generated in the study, which could influence how and where women use for birth. Women narrated that the placenta is regarded as the Ubioma (house of baby), “big baby” or “second baby” in Igala land. As many women recounted, the placenta is the first thing a woman will ask for after birth. A woman has not yet delivered if the placenta has not been handed to her. Igala’s believe that the placenta is linked to the baby’s whole life and destiny and influences how the baby turns out in the future, so if carelessly disposed of, could have negative influence on the growing baby. Most facilities in the context where the data was collected are critically aware of this cultural value and so normally hand over the placenta to families after birth, which also reinforces women’s continual use of such facilities around childbirth. However, women believe that inappropriately disposed placentas could be used for witchcraft or rituals that could negatively influence both the mother and baby and could lead to infertility in most cases. Thus, women narrated that the placenta is usually secretly buried by the child’s father or a trusted male after washing and wrapping the placenta with fresh, clean leaves. While burying the placenta, incantations and prayers are offered to maintain the link between the placenta and the baby. Based on the value placed on the placenta in Igala land, women narrated that they will never use or deliver in a facility where the workers do not hand over the placenta to them and that such behaviour will be communicated to other community members.

That is how I disposed of my own when I give birth in the hospital, my husband brings it back to where we live and then buries it there. Because most people believe that it should not be tampered with, that when the placenta is taken by maybe an animal or another person without you knowing, the destiny of that child can be averted. So that is why people guard it; when their wife gives birth, they take it, either the father of the child or the mother of the woman that gives birth takes it home and buries it so that it will be safe and another person will not take it, so that the life of the child and destiny of the child will be preserved (UA/FGD/01).

#### Rising matriarchs

While there are several cultural issues influencing women’s access to MHS, many women are rising above some of these cultural norms that are negatively influencing women’s use of MHS. These women are not rising to challenge the culture of the community but to empower other women and to create awareness of the need for women to use services provided by health facilities in the area. This new generation of women or rising matriarchs are empowered through education, mass media and years of childbearing experiences to encourage other women during informal gatherings, discussions, and talks on the need to embrace modern health services provided by health facilities. These women also look out for other pregnant women and friends, encouraging them to remember antenatal days and immunization days, checking on them when they are sick, and lending a helping hand whenever possible to ensure their friends, family, and colleagues remain healthy and use facility care throughout pregnancy.

Actually, like me, I use to tell my friends that the era of cultural belief when it comes to pregnancies and some things, has gone. Let us access and go to the correct places when we are pregnant… Like me, if I see any of my friends, I will ask them I hope you are attending a good clinic. We don’t want anything to happen to you or your baby. They always laugh and say you are always concerned. Yes, I am concerned; I don’t want anything to happen to my friends (RA/IDI/O1).

## Discussion

In this study, we have discussed many significant explicit and implicit cultural values, their meanings and how they influence women’s use of MHS among the Igalas in Nigeria. Using the relationships and the expectations domain of the PEN-3 cultural model provided a deep understanding of significant perceptions, enabler and nurturers that influence women’s use of MHS. As a negative perception, women’s belief in witchcraft significantly limited access to facility care and increased dependency on alternative Indigenous care approaches, which are believed to promote holistic healing when compared to facility care around childbirth. A similar finding was observed in Pakistan, Tanzania, Ghana, and South Africa [1, 45–48], where belief in witchcraft was believed to limit women’s access to MHS and influence the outcome of pregnancy. Belief in witchcraft drives women to seek protection from alternative and Indigenous care practices, which are believed to have potent power to ward off evil attacks [1].

Similar to the findings in this study, belief in witchcraft was also found to be the reason behind secrecy associated with early pregnancy and labour in Tanzania and South Africa, which influences access to antenatal and birth [47, 48]. Such lateness in accessing facility care could lead to late detection of preventable maternal health issues, resulting in maternal health complications. Given the challenges of late access to health facilities during pregnancy and labour, many authors have emphasized the need for maternal health awareness campaigns through social media within communities, which will create awareness of the importance of MHS [47, 48]. Studies reveal that such awareness could enhance the dissemination of maternal health information among women and within communities to promote knowledge and behaviour change toward appropriate use of MHS [48–51]. Moreover, the dissemination of maternal health information could be strengthened by developing maternal health education modules in health facilities that integrate diverse cultural norms to enhance women’s awareness of how such cultural norms could influence maternal health outcomes.

Home birth was also a significant theme found in the study, similar to findings in Ghana and Uganda [52–54], where many decide to deliver at home due to their socioeconomic status and inability to afford the cost of MHS. Lack of access to facility care due to poverty is a critical issue in Nigeria, which limits MHS access to many women of poor socioeconomic status [55]. While many studies have suggested free MHS as an incentive to enhance the use of health services, several studies [20, 21, 56] found that free MHS does not always imply that women would access facility care as several factors, such as cultural factors and hidden cost of transportation are limitations to access of health services. Thus, free MHS is crucial to enhance facility birth. However, further studies are needed to promote an in-depth understanding of how the hidden cost of MHS influences the use of such services when provided at no cost.

Furthermore, Adatara et al. [52] also found that psychological support and the need for access to culturally appropriate meals during birth and after drive women to deliver at home, similar to this study’s findings. Though many women in the present study understand the complications associated with home birth, such as bleeding, many were not discouraged from delivering at home. Women in the study narrated that their relationships and interactions within their homes and community promote a significant social support network that enhances alternative cheaper, supportive, culturally appropriate options around pregnancy and birth, which was also found in a study conducted in Uganda [54]. Women recounted that such social networks provide relational, emotional, financial, and physical support, mostly lacking in most health facilities. According to Mamo et al. [57], social support is a critical factor that needs to be explored in future maternal health interventions as such could enhance facility birth. However, the focus on social support should not be limited to general social support but should extend to support women receive continually throughout childbirth from family and close friends who play significant roles around childbirth [57].

In this study, we also found that the fear of cesarian section was one of the factors that limited women’s use of facility care. Women narrated that the cultural interpretation of cesarian section, fear of death, fear of not achieving a normal birth in subsequent births and fear of the doctor leaving a foreign object in their womb, among other reasons, scared women from accepting cesarian sections, also similar to the finding in Uganda and Ghana [58, 59]. However, contrary findings were reported in Thailand and Iran, where women openly requested cesarian sections due to reasons of a set date fixed for birth, safety, speed of birth, fear of labour pain and prestige [60, 61]. The difference between the findings of Shirzad et al. [61] and Suwanrath et al. [60] and the present study could be due to the fact that these studies were conducted in a context outside of SSA, where women have more exposure to health information. Cesarian section is a critical childbirth method which, when performed promptly, could limit maternal and fetal mortality. Given the contradictory findings in other contexts, diverse approaches tailored to the needs of each context are critical to enhancing positive maternal and fetal health outcomes.

While the birth of a child among the Igalas brings significant joy and happiness, the birth of the placenta (Ubioma) brings even greater joy and a delightful end to birth. The placenta is greatly valued in both communities where data was collected, as it is believed to have a unique link to the baby, invoking special rituals and burial. Such value placed on the placenta is also found in other countries, such as Ghana, Zambia, and Kenya, where the placenta is also valued and believed to be linked to the baby [62–64]. While the placenta may hold such values, according to Adatara [62], the placenta is inappropriately disposed of in some health facilities in Ghana. In the communities where the study was conducted, the placenta is carefully handed over to the families for culturally appropriate ritual and subsequent burial, which allows families to reconsider using such facilities in future pregnancy and birth. Based on the significance of the placenta in most SSA countries, understanding people’s cultural expectations and integrating such into care through providing culturally appropriate care is essential to promoting women’s use of facility care. Additionally, integrating Indigenous care practices into MHS has enhanced holistic care practices that promote health outcomes in many countries [65–67]. However, challenges to the transparency of Indigenous medical practice in most SSAs hinder such integration [68]. Consequently, understanding approaches to integrating Indigenous care into the health system, which is lacking in Nigeria, is crucial and could enhance collaboration within both health systems.

Providing respectful and culturally appropriate care is grounded on cultural awareness and sensitivity, which is established with understanding and learning about other cultures and the beliefs associated with such cultures [69, 70]. Cultural awareness recognizes that peoples’ values, beliefs, and assumptions are shaped by their culture, influencing how they perceive the world and relate to people around them [69, 70]. Thus, health workers must be aware of their cultural values and beliefs, which then enhances their understanding of the differences in other peoples’ values and beliefs [69]. Though there are limited culturally focused interventions in SSA and Nigeria, where the study was conducted, findings in Australia and Peru, where such culturally appropriate care was introduced among the Aboriginal population, showed increased use of MHS and satisfaction among women. Consequently, appropriate culturally focused interventions tailored to the maternal health needs of distinct populations are critical in SSA and in Nigeria to enhance women’s use of MHS and achievement of SDG #3:1.

The study’s findings show that the Igalas in Nigeria have a rich cultural heritage that is beneficial, protective, existential, and potentially harmful, influencing women’s access to MHS. These cultural patterns are intergenerational, deeply rooted, and accepted as a way of life. While many women who participated in the study have a quiet, respectful and accepting disposition concerning these cultural patterns influencing facility care, many women are rising, not to challenge the culture but to address some of the potentially harmful cultural patterns influencing women’s use of MHS through informal engagement with other women. Many of these rising matriarchs are women who are generally empowered by education. Education has been recognized as a propelling force promoting women’s use of MHS in many SSA countries, such as Uganda and Congo [71, 72]. The more years of education a woman acquires, the better chance a woman has to update herself with maternal health education and challenge cultural norms and traditions that are barriers to using contemporary MHS [71, 73, 74]. Consequently, education should be a critical focus in maternal health interventions and women empowerment programs in Nigeria and other countries in SSA.

## Strengths and limitations

This study was conducted using a focused ethnographic methodology, which allowed us to employ different methods such as participant observations, interviews, focus group discussions, field note and reflective memo to enhance a thick description and interpretation of our findings. Moreover, using the PEN 3 cultural model to organize our findings assisted in providing a detailed link between each construct of the relationships and the expectations domain of the PEN 3 cultural model and the findings in the study. Besides, using purposive and snowballing techniques allowed for the inclusion of women with diverse demographic characteristics that enriched the study. However, this study was confined to women accessing primary healthcare facilities, and so may not be generalized to other levels of care, such as secondary and tertiary health facilities in the country. Consequently, future research could focus on other levels of the health system in Nigeria.

## Policy statements

This study has enhanced a deep understanding of implicit and explicit cultural patterns influencing women’s use of MHS in Nigeria. Cultural issues related to the use of MHS is a multifaceted issue that requires collaboration between health systems and communities to enhance appropriate culturally focused MHS strategies that are tailored to the cultural needs of communities [75–77]. However, community engagement, which should be a significant part of the function of primary health facilities in Nigeria, is non-functional. Such engagement would allow facilities, religious groups, communities, and traditional leaders, who are custodians of culture and role models in the community, to deliberate on maternal health issues and formulate culturally appropriate approaches to ensure MHS are responsive to the cultural needs of communities. Consequently, stakeholders and policymakers must seek appropriate measures to reestablish community engagement in primary health facilities in Nigeria, to enhance maternal health outcomes.

In addition, the government should provide culturally competent training and modules for nurses and other health workers to enhance self-awareness and understanding of the culture of diverse ethnicities in Nigeria. Cultural competence ensures nurses and healthcare workers understand and respect the cultural beliefs, values, and practices of others, which could promote satisfaction and holistic care provision to diverse ethnicities in Nigeria [78].

Given the rich cultural heritage of diverse ethnicities in Nigeria, which generally influences decisions on the use of MHS around pregnancy and childbirth, policies should be focused on the provision of appropriate maternal health education booklets that integrate cultural values, beliefs, and practices surrounding childbirth that influence women’s use of MHS. Culturally focused maternal health education during antenatal, birth and postnatal care is essential to create awareness of beneficial, neutral, and harmful cultural values and practices that could influence the outcome of pregnancy, labour, or birth. Such health teachings are crucial to creating awareness and understanding of traditional values and practices, which are seen as protective, though some could be harmful and limit access to facility care. However, such knowledge acquisition is further influenced by women’s level of education, which is most favourable when women have a higher level of education [79]. Consequently, strategies and interventions should focus on approaches to tailor health information to educationally deprived populations, especially in rural areas of Nigeria, to ensure comprehensive coverage and understanding of health information. Additionally, the provision of free MHS in all states of Nigeria, especially in remote areas, where most cultural norms and practices are dominant, is significant to enhancing women’s use of MHS.

In summary, introducing culturally competent maternity care in healthcare is critical to promoting health workers’ understanding and acceptance of people’s cultures and their preferences in care, which will ensure satisfaction and holistic care provision [79]. Thus, introducing policies that ensure culturally appropriate care is critical to improving maternal health in Nigeria and realization of the SDG goal #3:1.

## Conclusion

This study aimed to explore explicit and implicit cultural patterns influencing women’s use of MHS in Nigeria. Using the focused ethnographic methodology described by Roper and Shapira (2000), we employed diverse methods such as interviews, focus group discussions, participant observations, field note and reflective memo, which enhanced a thick description of our findings. Our findings were organized using the relationships and expectations domain of the PEN-3 cultural model, which generated three themes: perceptions, enabler and nurturers. The subthemes that were generated under the perceptions were belief in witchcraft, which created immense fear and limited women’s use of MHS. The fear of witchcraft and attacks from evil people limited early access to MHS during pregnancy and labour. Additionally, a quiet, unspoken acquiescence to the culture also limited women’s decision-making abilities, even in the use of MHS. The subtheme generated under the theme of enabler was home birth, where support was provided for women at home, and financial issues limited women’s use of MHS. In the theme of nurturers, we found that the cultural interpretation of cesarian section creates fear and limits women’s access to facility care. Additionally, the value placed on the placenta and health workers’ approach to placenta disposal determines if such a facility would be accessed in subsequent deliveries. However, with diverse cultural factors influencing the use of MHS, many women in the communities are rising to ensure that cultural barriers do not limit women’s access to facility care through informal education. Given the deep-seated cultural issues that influence women’s use of MHS, a multi-faceted intervention is needed to ensure that MHS is tailored to the cultural needs of women to enhance maternal health outcomes and realization of SDG #3.1 in Nigeria.

## Electronic supplementary material

Below is the link to the electronic supplementary material.

Supplementary Material 1

Supplementary Material 2

Supplementary Material 3

## Data Availability

Data are available on the reasonable request from the corresponding author.

## Abbreviations

FE: Focused ethnography
FGD: Focus group discussion
IDI: In-depth interview
LGA: Local government areas
MHS: Midwives service scheme
RA: Rural area
SDG: Sustainable development goals
SRQR: The standard for reporting qualitative research
SSA: Sub-Saharan Africa
UA: Urban area
WHO: World health organization
