# Supplementary material for: Visible and invisible cultural patterns influencing women’s use of maternal health services among Igala women in Nigeria: a focused ethnographic study
Source: BMC Public Health. 2025 Jan 13;25:133. doi: 10.1186/s12889-025-21275-9 (PMC11727540; doi:10.1186/s12889-025-21275-9)
Supplement: Supplementary file 1 — Supplementary Material 1 [file 12889_2025_21275_MOESM1_ESM.pdf]

UNIVERSITY OF SASKATCHEWAN
College of Nursing
NURSING.USASK.CA

College of Nursing
Health Sciences Building A-Wing 1A10
107 Wiggins Road, Saskatoon, Saskatchewan S7N 5E5
Telephone: (306) 966-6221 Facsimile: (306) 966-6621

March 28, 2024

To Whom it May Concern,

Uchechi Opara is a full-time student enrolled in the Doctor of Philosophy in Nursing program in the College of Nursing at the University of Saskatchewan.

This letter is to confirm that Uchechi is not currently receiving research funding.

If you have any questions regarding this, please do not hesitate to contact me.

Sincerely,

Louise Racine

Louise Racine RN, PhD, FTNSS
she/her/
Professor
Associate Dean Research and Graduate Studies
University of Saskatchewan
College of Nursing
