# Supplementary material for: Visible and invisible cultural patterns influencing women’s use of maternal health services among Igala women in Nigeria: a focused ethnographic study
Source: BMC Public Health. 2025 Jan 13;25:133. doi: 10.1186/s12889-025-21275-9 (PMC11727540; doi:10.1186/s12889-025-21275-9)
Supplement: Supplementary file 2 — Supplementary Material 2 [file 12889_2025_21275_MOESM2_ESM.docx]

**Visible and Invisible Cultural Patterns Influencing Women’s Use of Maternal Health**

**Services in Nigeria: A Focused Ethnographic Study**

**Participants Interview Guide Document; (One-on-One Interview)**

**Preambles**

Introduce yourself to the participant.

Describe the purpose of the interview and how information will be used.

Obtain oral consent.

Provide instructions guiding the interview.

Interview place:

Interview date:

Start time:

End time:

**Information Sheet**

| Participant Identification |  |
| --- | --- |
| Age |  |
| Number of pregnancies |  |
| Number of births |  |
| Number of children |  |
| Educational level |  |
| Occupation |  |
| Family income |  |
| Religion |  |
| Place of residence |  |

**General Question:**

How has your day been today**?**

**Maternal Health Services**

**1.** Can you tell me your understanding of health services provided for women in primary health facilities?

**Probes:**

a. During pregnancy?

b. During labour?

c. After delivery?

**2.** What are the services provided for you in this facility?

**Probes:**

1. Services provided for you during pregnancy.
2. Services provided for you during delivery.
3. Services provided for you from the time of delivery to six weeks after delivery.

**3.** Traditionally, what beliefs and practices are passed on to you as Igala women around pregnancy and childbirth?

**Probes:**

1. Traditional beliefs and practices during pregnancy?
2. Traditional beliefs and practices during delivery?
3. Traditional beliefs and practices observed after delivery.

4. How do these beliefs and practices influence how you use maternal health services offered in this facility?

**Probes:**

1. During pregnancy?
2. During delivery?
3. After delivery?

**5.**  As an Igala woman, what are your views of traditional beliefs and practices influencing women’s use of maternal health services?

**Probe:**

1. Beliefs and practices influencing women’s use of maternal health services?

**6.** How do you think other women see these traditional beliefs and practices around pregnancy and childbirth and their influence on women’s use of maternal health services

7. What do these cultural beliefs and practices mean to you as an Igala woman?

**Probes**

1. Could you tell me the importance of these traditional beliefs and values to you as an Igala woman who is pregnant or giving birth?
2. How important are these traditional beliefs and practices to other Igala women who are pregnant or giving birth?

**7.** How long do you stay at home before registering for antenatal care in this facility when you notice you are pregnant?

**Probes:**

1. What makes you decide when to register for antenatal care in this facility?
2. When do other women register for antenatal care in this facility?
3. How does your tradition or culture influence when you register for antenatal care in a health facility during pregnancy?
4. What could be the importance of registration for antenatal care in a health facility?
5. What could be the disadvantages of registration for antenatal care in a health facility?

8. Could you tell me some of the cultural factors that determine when you go to the facility for delivery when you are in labour?

**Probes**

1. Could you tell me more about why you decide to go to the facility at the time you do during labour and delivery?
2. Who accompanies you to the facility when you are in labour?
3. What do you think could be the traditional reason behind women’s non-use of the facility for delivery?
4. What could be other reasons many women do not use the facility for delivery?

**Conclusion**

1. Are there other things that you would want to tell me that we have not discussed?
2. Thank and appreciate the participant.

**Guide for Focus Group Discussions**

**Preambles**

Introduce yourself to the participants.

Describe the purpose of the focus group discussion and how information will be used.

Obtain oral consent.

Provide instructions congruent with focus group discussion.

Focus group discussion context:

Focus Group discussion date:

Start time:

End time:

**Information Sheet**

| Participant Identification |  |
| --- | --- |
| Age |  |
| Number of pregnancies |  |
| Number of births |  |
| Number of children |  |
| Educational level |  |
| Occupation |  |
| Family income |  |
| Religion |  |
| Place of residence |  |

**General Question:**

How has your day been today**?**

**Maternal Health Services**

**1.** Can you tell me your understanding of health services provided for women in primary health facilities?

**Probes:**

a. During pregnancy?

b. During labour?

c. After delivery?

**2.** What are the services provided for you in this facility?

**Probes:**

1. Services provided for you during pregnancy.
2. Services provided for you during delivery.
3. Services provided for you from the time of delivery to six weeks after delivery.

**3.** Traditionally, what beliefs and practices are passed on to you as Igala women around pregnancy and childbirth?

**Probes:**

- 1. Traditional beliefs and practices during pregnancy?
  2. Traditional beliefs and practices during delivery?
  3. Traditional beliefs and practices observed after delivery.

4. How do these beliefs and practices influence how you use maternal health services offered in this facility?

**Probes:**

1. During pregnancy?
2. During delivery?
3. After delivery?

**5.**  As an Igala woman, what are your views of traditional beliefs and practices influencing women’s use of maternal health services?

**Probe:**

1. Beliefs and practices influencing women’s use of maternal health services?

**6.** How do you think other women see these traditional beliefs and practices around pregnancy and childbirth and their influence on women’s use of maternal health services

7. What do these cultural beliefs and practices mean to you as an Igala woman?

**Probes**

1. Could you tell me the importance of these traditional beliefs and values to you as an Igala woman who is pregnant or giving birth?
2. How important are these traditional beliefs and practices to other Igala women who are pregnant or giving birth?

**7.** How long do you stay at home before registering for antenatal care in this facility when you notice you are pregnant?

**Probes:**

1. What makes you decide when to register for antenatal care in this facility?
2. When do other women register for antenatal care in this facility?
3. How does your tradition or culture influence when you register for antenatal care in a health facility during pregnancy?
4. What could be the importance of registration for antenatal care in a health facility?
5. What could be the disadvantages of registration for antenatal care in a health facility?

8. Could you tell me some of the cultural factors that determine when you go to the facility for delivery when you are in labour?

**Probes**

1. Could you tell me more about why you decide to go to the facility at the time you do during labour and delivery?
2. Who accompanies you to the facility when you are in labour?
3. What do you think could be the traditional reason behind women’s non-use of the facility for delivery?
4. What could be other reasons many women do not use the facility for delivery?

**Conclusion**

1. Are there other things that you would want to tell me that we have not discussed?
2. Thank and appreciate the participant.
