# Supplementary material for: Visible and invisible cultural patterns influencing women’s use of maternal health services among Igala women in Nigeria: a focused ethnographic study
Source: BMC Public Health. 2025 Jan 13;25:133. doi: 10.1186/s12889-025-21275-9 (PMC11727540; doi:10.1186/s12889-025-21275-9)
Supplement: Supplementary file 3 — Supplementary Material 3 [file 12889_2025_21275_MOESM3_ESM.docx]

**Visible and Invisible Cultural Patterns Influencing Women’s Use of Maternal Health**

**Services Among Igala Women in Nigeria: A Focused Ethnographic Study**

# *Uchechi Clara Opara^*1^, Peace Njideka Iheanacho^2^_,_ Pammla Petrucka^1^*

College of Nursing, University of Saskatchewan, Canada^1^.

Department of Nursing Sciences, University of Nigeria, Enugu Campus^2^

**Authors Details**

Uchechi C. Opara

Doctoral Candidate at the College of Nursing, University of Saskatchewan, Health Science Building - 1A10, Box 6, 107 Wiggins Road, Saskatoon, SK S7N 5E5​. Saskatchewan, Canada.

Phone: +1306 281 3986

Email Address: uco020@usask.ca (Corresponding Author)

Peace Njideka Iheanacho

Associate Professor at the Department of Nursing Sciences, University of Nigeria, Enugu Campus, Enugu State, Nigeria.

Phone: +2348086774004

### Email Address: peace.iheanacho@unn.edu.ng

Pammla Petrucka

Professor at the College of Nursing, University of Saskatchewan, Health Science Building-1A10, Box 6, 107 Wiggins Road, Saskatoon, SK S7N 5E5​, Saskatchewan, Canada.

Phone Number: +13063373800

Email Address: pammla.petrucka@usask.ca

**Author Details**

^1^ UCO is a doctoral candidate at the College of Nursing, University of Saskatchewan, Canada

^2^ PNI is an associate professor at the Department of Nursing Sciences, University of Nigeria, Enugu Campus

^3^ PP is a professor at the College of Nursing, University of Saskatchewan, Canada
